# The Quantum-Medical Nexus: Understanding the Impact of Quantum Technologies on Healthcare

**DOI:** 10.7759/cureus.48077

**Published:** 2023-10-31

**Authors:** Muhammad Shams, Jinal Choudhari, Katherine Reyes, Sophia Prentzas, Abubakar Gapizov, Abdullah Shehryar, Maryam Affaf, Han Grezenko, Rayan W Gasim, Syed Naveed Mohsin, Abdur Rehman, Shehryar Rehman

**Affiliations:** 1 Urology, Royal Bornemouth Hospital, Bournemouth, GBR; 2 Family Medicine, Division of Research and Academic Affairs, Larkin Community Hospital, Miami, USA; 3 Psychiatry, American University of Antigua, Osbourn, ATG; 4 Internal Medicine, American University of Antigua, Osbourn, ATG; 5 General Surgery, American University of Antigua, Osbourn, ATG; 6 Internal Medicine, Allama Iqbal Medical College, Lahore, PAK; 7 Internal Medicine, Women’s Medical and Dental College, Abbottabad, PAK; 8 Translational Neuroscience, Barrow Neurological Institute, Phoenix, USA; 9 Internal Medicine, University of Khartoum, Khartoum, SDN; 10 Orthopeadics, St. James Hospital, Dublin, IRL; 11 General Surgery, Cavan General Hospital, Cavan, IRL; 12 Surgery, Mayo Hospital, Lahore, PAK; 13 Internal Medicine, Al Assad University Hospital, Damascus, SYR

**Keywords:** historical milestones, communication, sensing, quantum computing, quantum mechanics, narrative review, healthcare, quantum technologies

## Abstract

In a world characterized by rapid technological evolution, the integration of quantum technologies into the realm of healthcare has emerged as a transformative force. This narrative review explores the journey of quantum innovations in medicine, delving into the fundamental principles of quantum mechanics that underpin quantum computing, sensing, and communication. From the birth of quantum theory to the advent of practical quantum applications, we journey through historical milestones that have paved the way for a quantum-powered future in healthcare.

The narrative unfolds to reveal the profound implications of quantum technologies in healthcare, ranging from accelerated drug discovery and genomic analysis to secure data transmission and telemedicine. Real-world case studies illuminate successful applications, while the review addresses the ethical, societal, and regulatory considerations that accompany this quantum revolution. As we peer into the future, we contemplate the challenges that lie ahead and offer recommendations for researchers and policymakers to forge a harmonious and equitable synergy between quantum and medicine. In a world where innovation outpaces the tick of the clock, this narrative review serves as a timely guide for those poised to shape the quantum healthcare landscape, where precision and compassion converge and the possibilities are limitless.

## Introduction and background

Quantum technologies utilize the principles of quantum mechanics, the branch of physics that describes the behavior of matter and energy at extremely small scales - on the order of atoms and subatomic particles [1]. Concepts like superposition and entanglement, native to quantum mechanics, have enabled the development of practical applications such as quantum computing, quantum sensing, and quantum cryptography [2].

The intersection of quantum technologies with medicine and healthcare has the potential to revolutionize various aspects of diagnosis, treatment, and data security [3]. For example, quantum computing could significantly accelerate the process of drug discovery by efficiently simulating molecular interactions [4]. Quantum sensors may offer unprecedented sensitivity in diagnostic procedures, allowing for the detection of biomarkers at previously unattainable levels [5]. Additionally, the use of quantum cryptography could enhance the security of medical records and facilitate truly private communication between healthcare providers and patients.

Given the maturation of quantum technologies and their growing relevance to healthcare, there is a pressing need for comprehensive literature that outlines the current state and prospects of this interdisciplinary field [6]. Despite promising advances, a significant gap exists in academic discourse that could potentially hinder the adoption and further innovation of quantum technologies in medicine. Therefore, a narrative review that consolidates existing research, examines applications and suggests future research directions is both timely and crucial.

The primary aim of this narrative review is to provide an exhaustive overview of the current applications and prospects of quantum technologies in medicine. This review will focus on how quantum computing, sensing, and cryptography are currently being applied or have the potential to be applied in drug discovery, diagnosis, personalized treatment planning, and data security. The ethical and societal implications of integrating quantum technologies into healthcare will also be explored, along with challenges and limitations that must be addressed [7].

## Review

Historical perspective

The Birth of Quantum Theory

The foundations of quantum theory can be traced back to the early 20th century when eminent physicists, including Max Planck, Albert Einstein, Niels Bohr, and Werner Heisenberg, embarked on a profound exploration of the fundamental principles of quantum mechanics [8]. This pivotal era marked a departure from classical physics as they delved into the intricate world of quantum phenomena. Max Planck's groundbreaking work in 1900, which introduced the concept of energy quantization, ushered in the quantum revolution by challenging the classical notion of continuous energy flow [9]. Albert Einstein further solidified quantum theory's emergence with his 1905 explanation of the photoelectric effect, revealing light's dual nature as both wave and particle [10]. These scientific pioneers sowed the seeds of quantum mechanics, initiating a transformative journey that would ultimately reshape our comprehension of the universe at its most fundamental level.

Early Quantum Technologies

In the nascent stages of quantum theory, its practical applications were largely confined to the realm of theory, as the foremost focus of physicists rested on unraveling the intricacies of its underlying principles. These pioneers of quantum mechanics were driven by an insatiable curiosity to decipher the enigmatic behavior of matter and energy at the smallest scales, pushing the boundaries of scientific understanding. However, as the mid-20th century emerged on the horizon, the profound insights gleaned from quantum mechanics began to crystallize into tangible innovations that would shape the course of scientific progress.

It was during this transformative period that early quantum technologies began to materialize, marking a pivotal transition from theoretical abstraction to practical realization. Among these pioneering advancements stood nuclear magnetic resonance (NMR) spectroscopy, a testament to the profound implications of quantum theory in the realm of scientific inquiry and medicine alike [11]. NMR spectroscopy harnessed the quantum properties inherent to atomic nuclei, opening doors to a new dimension of precision and sophistication in probing the molecular structures of matter. Initially conceived as a tool for unraveling the mysteries of fundamental physics and chemistry, NMR spectroscopy soon transcended its origins to become a cornerstone of medical diagnostics and the exploration of intricate biological processes. The journey from theoretical contemplation to tangible innovation had commenced, and the convergence of quantum mechanics with the field of medicine embarked on an extraordinary trajectory of discovery and progress.

Transition to Medical Applications

The transition from theoretical quantum physics to practical applications in medicine began in the latter half of the 20th century. One notable milestone was the development of magnetic resonance imaging (MRI), which relied on NMR principles [12]. MRI revolutionized medical imaging by providing detailed non-invasive views of internal structures, enhancing diagnosis and treatment planning.

Seminal Works and Milestones

Seminal works at the intersection of quantum technologies and medicine have notably revolved around the pioneering development of quantum sensors tailored for biomarker detection [13]. Quantum sensors, harnessing the intrinsic quantum phenomena of entanglement and superposition, have demonstrated an extraordinary capacity for detecting disease-associated biomarkers at the most incipient stages, showcasing unprecedented sensitivity [14]. These breakthroughs have forged a path towards a quantum-enabled frontier in medical diagnostics, where the fusion of quantum principles with cutting-edge sensor technology promises to revolutionize early disease detection and ultimately improve patient outcomes. Following are a few instances:

Quantum dots in cancer detection: Quantum dots, which are semiconductor nanocrystals, have been used as fluorescent probes in bioimaging. Due to their unique optical properties, they can be engineered to bind to specific cancer biomarkers. Some studies have demonstrated the use of quantum dots to target and visualize HER2, a breast cancer biomarker, with remarkable sensitivity.

Diamond quantum sensors for magnetic imaging: Nitrogen vacancy (NV) centers in diamonds have been harnessed as sensitive quantum sensors for magnetic imaging at the nanoscale. This principle has been applied to detect neural signals, which could indirectly highlight neurological diseases.

Quantum computing in medicine

Introduction to Quantum Computing

Quantum computing stands at the forefront of computational innovation, capitalizing on the intrinsic principles of quantum mechanics to process information in a manner fundamentally distinct from classical computing paradigms. At the heart of quantum computing's prowess lies two remarkable phenomena - superposition and entanglement - bestowing upon it the transformative capability to perform intricate calculations exponentially faster than its classical counterparts [2]. In the context of medicine, this quantum marvel emerges as a beacon of promise, poised to tackle the most intricate challenges in fields such as drug discovery, genomics, and the realization of personalized medicine. The quantum horizon beckons, offering a realm where the convergence of cutting-edge technology and healthcare holds the potential to redefine the boundaries of what is achievable in the quest for improved healthcare solutions.

Drug Discovery

Quantum algorithms are being developed to simulate molecular interactions with unprecedented accuracy and efficiency [4]. Quantum computers can model the behavior of molecules at the quantum level, enabling pharmaceutical researchers to identify potential drug candidates and predict their interactions with biological targets. This has the potential to significantly accelerate drug discovery processes.

Genomic Analysis

The landscape of genomic analysis finds itself on the brink of a transformative epoch, one characterized by the impending integration of quantum computing - a computational paradigm renowned for its prowess in handling voluminous datasets inherent in gene sequencing and genomic data processing [13]. Within this realm of quantum computation, the promise of expedited computational tasks looms large, encompassing critical processes such as sequence alignment, variant calling, and protein structure prediction. It is this acceleration, propelled by quantum algorithms, that holds the potential to usher in a new era of profound insights into genetics and the intricate mechanisms underpinning diseases.

Quantum computing, with its fundamental departure from classical computing, brings forth a formidable arsenal of computational power that defies the constraints of classical systems. In the context of genomic analysis, the implications of this quantum advantage are profound. Tasks that once demanded formidable computational resources and time, such as the alignment of genetic sequences, can now be executed with unprecedented speed and efficiency. Quantum algorithms, fine-tuned to the nuances of genomics, offer the tantalizing prospect of expeditiously deciphering the genetic code, enabling variant calling with heightened accuracy, and accelerating the prediction of complex protein structures.

Personalized Medicine

Quantum computing offers the prospect of tailoring medical treatments to individual patients through the use of quantum algorithms [15]. By analyzing a patient's genetic and health data with high precision, personalized treatment plans can be developed, optimizing therapeutic outcomes while minimizing side effects.

Case Studies

Concrete illustrations of quantum computing's impact on medicine emerge through compelling case studies, spotlighting the remarkable successes born from the synergy of quantum technology and healthcare. Notably, in the domain of drug discovery, quantum algorithms have accelerated the identification of promising drug candidates for formidable medical adversaries such as cancer and Alzheimer's disease [16]. Quantum computing's capacity to expedite the intricate process of simulating molecular interactions offers a glimpse into a future where drug discovery combines unprecedented speed with unparalleled precision. Moreover, quantum-powered genomic analysis has unraveled the mysteries of rare genetic disorders, illuminating their underlying mechanisms with newfound precision and depth of insight. These case studies illuminate how the fusion of quantum computing with medicine transcends theory, ushering in a powerful force that advances our understanding of complex diseases and the development of more effective therapies.

Limitations and Challenges

While quantum computing holds immense potential, it also faces significant challenges. Quantum computers are still in the experimental stage, and large-scale, fault-tolerant quantum machines are needed for practical medical applications [3]. Additionally, quantum algorithms and software development are areas of active research, and translating theoretical potential into practical solutions poses technical and computational challenges.

Quantum sensing and imaging

Introduction to Quantum Sensing and Imaging

Quantum sensing and imaging represent a paradigm shift in the realm of sensing technologies, harnessing the inherent and remarkable properties of quantum mechanics to augment their sensing capabilities significantly. Quantum systems, operating on principles such as superposition and entanglement, possess an innate capacity for achieving unprecedented levels of sensitivity and precision - a transformative attribute that aligns them seamlessly with the intricate demands of medical applications [17]. The advantages rendered by quantum sensing and imaging extend a broad spectrum of benefits to the field of medicine, encompassing the realms of diagnostics and beyond, where improved resolution and accuracy serve as beacons of progress, guiding the way toward a future where healthcare reaches new heights of precision and effectiveness.

Quantum Sensors in Diagnosis

Quantum sensors have found an increasingly pivotal role within the healthcare landscape, demonstrating their prowess in the detection of biomarkers, monitoring cellular activities, and an array of diagnostic functions [18]. Their unique capability to discern even the most minuscule changes within biological systems has ushered in a new era of healthcare, characterized by early disease detection and the precise monitoring of treatment efficacy. Quantum sensors, with their heightened sensitivity and precision, have transcended the limits of conventional sensors, illuminating a path toward medical diagnostics that is characterized by both unprecedented accuracy and timely intervention. In the realm of healthcare, where early detection can often be the linchpin for effective intervention, quantum sensors offer a transformative advantage. The following instances demonstrate the current utilization of quantum sensors in healthcare:

Quantum dots for bioimaging: Quantum dots have been successfully employed in imaging techniques due to their unique fluorescent properties. For instance, some studies have utilized quantum dots to trace specific proteins within cells, aiding in early disease detection and a better understanding of cellular processes.

Quantum-enhanced magnetocardiography: Detecting the heart's magnetic field can provide valuable insights into cardiac health. Quantum sensors using superconducting quantum interference devices (SQUIDs) have been employed in magnetocardiography studies to offer high-resolution imaging of cardiac magnetic fields.

Quantum Imaging Technologies

Within the realm of medicine, quantum-enhanced imaging technologies have emerged as formidable assets, promising to redefine the landscape of medical diagnostics and visualization [19]. Quantum entanglement, a remarkable phenomenon in quantum physics, has been harnessed to usher in a new era of medical imaging, characterized by improved resolution and enhanced contrast. These advancements, underpinned by the profound principles of quantum mechanics, are poised to revolutionize established techniques like MRI, where the precision and clarity of quantum entanglement render a transformative touch to the field. The fusion of quantum mechanics with medical imaging holds the promise of not only elevating the quality of diagnoses but also unraveling hitherto concealed intricacies within the human body, further propelling the frontier of healthcare visualization, and understanding.

Applications in Various Medical Fields

Quantum sensing and imaging find applications in a range of medical fields. In neurology, for instance, quantum sensors can detect subtle brain activity changes, aiding in the diagnosis and monitoring of neurological disorders [20]. Similarly, in cardiology and oncology, quantum-enhanced imaging techniques provide valuable insights into heart conditions and cancer detection. Following are specific examples highlighting the neurologic disorders where quantum sensors have shown promise:

Alzheimer's disease: Quantum sensors have shown potential in detecting early biomarkers for Alzheimer's Disease. For instance, by leveraging quantum dots, researchers have been able to target and image beta-amyloid plaques in neural tissues, a hallmark of Alzheimer's pathology.

Parkinson's disease: Magnetic sensing using diamond-based quantum sensors (with nitrogen vacancy centers) has been explored to study neural patterns in models of Parkinson's Disease. These sensors can detect minute magnetic fields produced by neural activity, thereby providing insights into dysregulated neural circuits seen in Parkinson's.

Case Studies

Real-world applications highlight the success of quantum sensing and imaging in medicine. For example, in neurology, quantum sensors have been used to detect biomarkers associated with Alzheimer's disease, enabling early diagnosis and intervention [21]. In cardiology, quantum-enhanced imaging has improved the visualization of cardiac structures and abnormalities.

Limitations and Challenges

Despite their promise, quantum sensing and imaging face limitations and challenges. Practical implementation often requires highly controlled environments, making them less accessible in certain medical settings [22]. Additionally, scaling up quantum technologies for widespread clinical use remains a significant challenge.

Quantum communication in healthcare

Introduction to Quantum Communication

Quantum communication, rooted in the foundational principles of quantum mechanics, emerges as the vanguard of secure and private data transmission. At its core, quantum cryptography, propelled by the enigmatic quantum properties of entanglement and superposition, forges a robust foundation for unassailable encryption methodologies [22]. Within the context of healthcare, this quantum marvel stands as a bulwark of security, safeguarding sensitive patient data and confidential medical information. The promise of quantum communication lies not only in its formidable encryption capabilities but also in its potential to revolutionize secure healthcare communication, ensuring that patient privacy remains sacrosanct in an increasingly interconnected world.

Secure Data Transmission

Quantum cryptography plays a crucial role in securing medical records and sensitive patient data. Quantum key distribution (QKD) protocols enable the exchange of encryption keys with absolute security, preventing eavesdropping and data breaches [23]. This technology is paramount in safeguarding patient privacy and medical information.

Telemedicine

Quantum-secured communication networks have immense potential in the field of telemedicine. Quantum-secured telecommunication infrastructure ensures that medical data transmitted between healthcare providers and patients remains confidential and tamper-proof [24]. This is especially critical for remote healthcare consultations and the exchange of vital patient information.

Case Studies

Real-world examples showcase the implementation of quantum-secured communication systems in healthcare. Hospitals and medical facilities are increasingly adopting quantum cryptography to protect electronic health records (EHRs) [25]. Telemedicine platforms that rely on quantum-secured networks are becoming more prevalent, ensuring the confidentiality of remote consultations and patient data.

Limitations and Challenges

Quantum communication in healthcare faces several limitations and challenges. Deploying and maintaining quantum-secured networks can be costly and complex [26]. Additionally, the development of practical quantum communication systems for widespread use requires overcoming technical hurdles and ensuring compatibility with existing healthcare infrastructure.

Ethical and societal implications

Data Security and Ethics

The advent of quantum-enabled healthcare ushers in a new era laden with critical ethical considerations, paramount among them being the safeguarding of data security. Quantum cryptography heralded for its impervious protection of medical records and patient information, stands as a formidable bulwark against unauthorized access and data breaches [27]. Yet, this remarkable promise of security is accompanied by an equally pressing responsibility - a responsibility to navigate the ethical intricacies inherent to the utilization of quantum technologies in healthcare. The ethical landscape unfolds to reveal multifaceted dimensions, encompassing the prevention of unauthorized access, the mitigation of data breaches, and the conscientious handling of quantum-secured patient data.

At the heart of quantum-enabled healthcare lies the ethical imperative to ensure that the impenetrable shield of quantum cryptography is wielded responsibly. Unauthorized access to quantum-secured data, while exceedingly challenging, remains a potential concern, necessitating stringent safeguards to deter malevolent intent. The specter of data breaches, while diminished, demands ongoing vigilance to preserve patient privacy and confidentiality. Moreover, the responsible stewardship of quantum-secured patient data becomes an ethical linchpin, with stringent protocols and policies required to maintain the integrity of sensitive medical information. As the quantum era dawns upon healthcare, the ethical compass of the medical community must remain steadfast, diligently navigating the complex terrain of data security and privacy, ensuring that the promise of quantum technologies is met with an unwavering commitment to ethical responsibility.

Accessibility and Equity

Ensuring equitable access to quantum medical technologies presents a significant challenge. The cost of developing and maintaining quantum-enabled healthcare systems can be prohibitive, potentially leading to disparities in healthcare access [28]. Addressing this issue requires careful consideration of affordability and accessibility for all patient populations.

Regulatory Concerns

The integration of quantum technologies into healthcare necessitates regulatory oversight. Healthcare regulatory bodies, such as the FDA, must adapt to evaluate the safety and efficacy of quantum-enabled medical devices and therapies [29]. Policy implications, standards, and guidelines for quantum applications in healthcare need to be developed and enforced to safeguard patient well-being.

Future prospects and recommendations

Upcoming Research and Technologies

The future of healthcare stands at the threshold of exciting possibilities, propelled by ongoing research in quantum technologies that portend transformative prospects. Quantum computing, sensing, and communication, advancing at an exhilarating pace, are poised to unveil a new era of healthcare innovation, with quantum algorithms primed to revolutionize complex medical simulations and diagnostics [30]. As the quantum realm intertwines with the medical domain, researchers are embarking on explorations into quantum machine learning and artificial intelligence applications, promising potential breakthroughs in data analysis and the realization of personalized medicine [31]. The horizon beckons with the allure of quantum-driven healthcare solutions, where the precision of quantum technologies converges with the intricacies of medical science, opening vistas of discovery and progress yet uncharted. Following is a more detailed account of the potentials of quantum technologies in healthcare:

Drug discovery: Quantum computers have the potential to simulate complex molecular and chemical reactions with high precision. This could lead to faster and more efficient drug discovery processes. For instance, understanding protein folding or simulating the interactions between drugs and their target molecules can be significantly expedited with quantum algorithms.

Genomic analysis: Quantum algorithms can assist in analyzing vast genomic data sets more efficiently. This could lead to personalized medicine tailored to an individual's genetic makeup and the prediction of disease susceptibility.

Improved medical imaging: Quantum-enhanced imaging techniques, such as those based on entangled photon pairs, can lead to better resolution and more precise images, which are especially crucial for diagnosing conditions at early stages.

Neural network training: Quantum machine learning can potentially train deep neural networks faster, refining the predictive models used in diagnostic procedures, and treatment plan optimization.

Challenges and Limitations

While the future is promising, quantum healthcare faces multifaceted challenges. Technical hurdles, such as scaling quantum systems for practical applications, remain a primary concern [32]. Ethical considerations surrounding data privacy and responsible use of quantum technologies persist. Moreover, regulatory frameworks need to adapt to accommodate quantum-enabled medical devices and therapies [33].

Recommendations for Researchers

Researchers in quantum healthcare should prioritize interdisciplinary collaboration. Combining expertise in quantum physics, medicine, and data science is crucial for advancing the field. Furthermore, a focus on developing robust and scalable quantum technologies suitable for medical settings is essential. Research efforts should also emphasize the ethical implications of quantum healthcare and explore strategies for responsible innovation [34].

Recommendations for Policy Makers

Policymakers play a vital role in creating an enabling environment for quantum healthcare. They should consider establishing regulatory frameworks that ensure the safety and effectiveness of quantum-enabled medical technologies while promoting equitable access. Supporting research and development in quantum healthcare through funding and incentives is vital. Policymakers must also engage with stakeholders to address ethical concerns and privacy issues, safeguarding patient rights and data security [35].

## Conclusions

In our ever-evolving world, the pace of technological advancement has reached unprecedented heights, ushering in a new era of possibilities in healthcare. Quantum technologies, rooted in the profound principles of quantum mechanics, have emerged as a beacon of innovation, illuminating the path toward a future where medicine is more precise, secure, and accessible than ever before. As we stand at the intersection of quantum and healthcare, we find ourselves on the precipice of transformative change.

This narrative review has endeavored to encapsulate the extraordinary journey of quantum technologies in medicine, from their nascent beginnings to the cusp of revolutionizing diagnosis, treatment, and data security. The rapid convergence of quantum computing, sensing, and communication with healthcare has opened vistas of potential. Yet, with boundless potential comes a profound responsibility. The ethical and societal implications, the challenges that await, and the recommendations for a harmonious fusion of quantum and medicine have all been contemplated. In this fast-paced world, where new technologies unfurl at a breathtaking pace, this article stands as a timely chronicle, a guidepost for those who seek to navigate the quantum frontier in healthcare. Together, as highly qualified medical professionals, researchers, and policymakers, we carry the mantle of shaping this quantum future, where precision meets compassion, and innovation knows no bounds.
